# Subspecialisation in Urology: Setting standards or creating monopoly?

**DOI:** 10.4103/0970-1591.42604

**Published:** 2008

**Authors:** Nitin S. Kekre, Chandra Singh J.

**Affiliations:** Department of Urology, Christian Medical College, Vellore - 632 004, Tamilnadu, India. E-mail: editor@indianjurol.com

This issue comes with two letters that address this crucial question. Both focus on the core issue – the pediatric patient with urological problems. First, we need to resolve if we are interested in delivering the best possible care for every child or to somehow deliver reasonable care for everyone. If our objective is the former, we need to aim for the best available standard of care and that requires a well-orchestrated effort and commitment. If we resolve to be satisfied at the latter, we probably do not need to progress much.

Another aspect that needs to be addressed is training and who can be trained best to subspecialize in pediatric urology. Though areas like embryology and neonatal physiology are essential, they can be learnt, as how a pediatric surgical trainee learns during his/her training. Other aspects like evaluation of pediatric patient and intraoperative tissue handling, if not already exposed during urology training, can be learnt during a posting in pediatric surgery. But the training in adult urology obtained by a urology trainee enables him or her to understand the complex pathophysiology of voiding, long-term impact of bowel substitution etc. that play a crucial role in intraoperative decision-making. A urology trainee encounters the impact of neonatal and pediatric interventions in patients of several age groups. He/she continues to see them in adulthood. Hence, one will not flippantly decide to augment the bladder or make radical decisions without having objectively studied the disease process precisely prior to intervention.

When we recognize that the American model is worth emulating for the standard of care, lack of infrastructure should not be given as the reason for suggesting an inferior alternative. It is possible to provide comparable standard of care and we have achieved this in India in several specialties, including uro-oncology, transplantation, and minimally invasive surgery. It is evident from the blooming health tourism that we can give comparable, if not better care. The possible need for more personnel to care for the geriatric population is an interesting speculation. But the impact of such a possibility has not been evident from the developed countries that had a downward trend in birth rate several decades back. Moreover, with our total population heading toward numero uno status, there will be no dearth of patients with anomalies that occur with predictable incidence rates. Furthermore, with the antenatal care staggering far behind our population growth, we will continue to see exstrophies and meningomyeloceles, and the need for developing expertise and centers of excellence will not be questioned against the backdrop of economic development.

Commercial viability of subspecialties depends primarily upon the quality of care. Though such an apprehension was prevalent when specialties of surgery were thought of, trained urologists and general pediatric surgeons in the present era have enough volume of patients. Similarly, this will cease to be an issue when pediatric urology is established as a subspecialty. This is evident from the countries where this has been accomplished in pediatric urology.

Congenital and chromosomal anomalies do involve multiple systems. But as much as a cardiac surgeon needs to be involved in the care, urogenital and colorectal involvement requires the combined input of the pediatric urologist and pediatric colorectal surgeon. Urogenital system alone should not be given step-motherly care just because of the easy accessibility and proximity to other organs being treated.

Though ability to perform upper tract endoscopy or renal transplantation alone cannot be the defining parameter, performing a pyelolithotomy in a child who is prone to recurrent stone episodes cannot be justified when one is talking of quality care. The claim that the current generation of pediatric surgeons is adequately trained to treat common and critical urological problems needs to be substantiated. Furthermore, it requires more than managing the common and critical problems, to become a pediatric urologist.

It is indeed deplorable to infrequently perform complex reconstructions resulting in high complication rate. This underscores the need for establishing pediatric urology as a subspecialty that will reflect on our outcome.

Neither organ-based specialists nor general pediatric surgeons can do justice to pediatric urology. It requires dedicated training in pediatric urology to understand the complexities and embryobiology of the urogenital system as well as the variations of neonatal and pediatric pathophysiology if we need to do justice to children with urological problems. We need to apply the motto of the British Association of Paediatric Surgeons when describing the role of pediatric urology: to set a standard, not to create a monopoly.

This issue's symposium focuses on genitourinary tuberculosis (GUTB) and has covered several important areas. Recent advances from investigative and therapeutic aspects of tuberculosis have been incorporated. With the high prevalence of tuberculosis and protracted morbidity of GUTB, this symposium will come in handy to address several issues. We are indeed thankful to Prof. Narmada Gupta and his colleagues for their contributions. We have always felt that, though the urologists of Asian continent treat a substantial number of patients with GUTB, we have had relatively scant publications or research on this topic. We sincerely hope that this symposium would stimulate all Asian colleagues to report on long-term data and undertake further clinical research.

Several interesting review articles have been incorporated in this issue. Philip Dahm has kindly consented to be in the international advisory committee of IJU and his team has contributed a couple of articles on surgical trials. Understanding evidence-based medicine is essential for good clinical practice and an understanding of evidence-based urology is important before we judge the quality of any literature.

One of the commonest dilemmas in practice is to differentiate between nocturia and nocturnal polyuria. Moon et al have addressed this in their article. In uro-oncology, we have spanned between prostate cancer prevention and management advances in hormone refractory prostate cancer. A new section on point-counterpoint has been included from this volume. The first debate addresses the adequacy of urology training.

As this volume is being released before the Asian Congress of Urology, we extend a hearty welcome to the participants of the conference. We invite submissions to our journal from the participants and we are committed to prompt processing and publication of accepted manuscripts. As you enjoy the Indian hospitality, may the Indian recipes and colors leave lasting memories!

